# Efficient Lung Cancer Molecular Diagnostics by Combining Next Generation Sequencing with Reflex Idylla Genefusion Assay Testing

**DOI:** 10.3390/genes14081551

**Published:** 2023-07-28

**Authors:** Dingani Nkosi, Giby V. George, Huijie Liu, Meghan Buldo, Moises J. Velez, Zoltán N. Oltvai

**Affiliations:** Department of Pathology and Laboratory Medicine, School of Medicine & Dentistry, University of Rochester, Rochester, NY 14642, USA

**Keywords:** lung cancer, molecular diagnostics, next-generation sequencing, Idylla platform

## Abstract

Molecular diagnostics for lung cancer is a well-established standard of care, but how to use the available diagnostic tools for optimal and cost-effective patient care remains unresolved. Here, we show that DNA-only, small gene next-generation sequencing (sNGS) panels (<50 genes) combined with ultra-rapid reflex testing for common fusion transcripts using the Idylla Genefusion assay provide a cost-effective and sufficiently comprehensive testing modality for the majority of lung cancer cases. We also demonstrate the need for additional reflex testing capability on larger DNA and fusion panels for a small subset of lung cancers bearing rare single-nucleotide variants, indels and fusion transcripts and secondary, post-treatment resistance mutations. A similar testing workflow could be adopted for other solid tumor types for which extensive gene/fusion variant profiles are available both in the treatment-naïve and post-therapy settings.

## 1. Introduction

More than 65% of patients diagnosed with advanced non-small cell lung cancer harbor actionable gene mutations, gene amplifications, or oncogenic fusion transcripts [1,2]. Consequentially, NGS-based tumor molecular profiling is now the standard of care for patients with advanced non-small cell lung cancer (NSCLC) [3,4,5,6]. Multiple genetic alterations have been identified as therapeutic targets in lung adenocarcinoma, including mutations in *EGFR*, *ERBB2*, *BRAF*, *KRAS*, and *MET* and rearrangements of *ALK*, *RET*, and *ROS1* [4,5,7,8]. Moreover, several other oncogenes with potential prognostic roles in lung adenocarcinoma, including *MET* and *PIK3CA*, have also been described [7,8,9]. Currently, various diagnostic tools and platforms are being used in both academic and commercial pathology laboratory settings to profile lung tumors. The modalities being used include fluorescence in situ hybridization (FISH), immunohistochemistry (IHC), and next-generation sequencing (NGS). However, the optimal combination(s) of the different diagnostic modalities to enhance timely yet cost effective patient care remains to be determined.

Currently, as is the case in most academic laboratories, the University of Rochester Medical Center (URMC) molecular genetic pathology (MGP) unit (molecular diagnostics and cytogenetics laboratories) utilizes both FISH and NGS to profile actionable genomic mutations and rearrangements in lung cancer. In the present URMC workflow, parallel testing for *ALK* and *ROS1* rearrangements is evaluated through FISH and the search for gene mutations and amplifications is conducted using a <50 gene NGS panel at the DNA level only (Oncomine Focus Assay [OFA], see Methods). In addition, the URMC anatomic pathology laboratory performs IHC-based testing for *ALK* and *ROS1* fusions and for *NTRK 1/2/3* fusions (using a panNTRK antibody), respectively.

Here, we reevaluate this workflow for lung cancer molecular diagnostics. We first describe the validation of the Biocartis Idylla Genefusion assay for detection of *ALK*, *ROS1*, *RET* fusions, and *MET* exon 14 skipping mutations, which is performed at the RNA level on tissues obtained directly from formalin-fixed paraffin embedded (FFPE) slides requiring less than 5 min hands-on time from a technologist. We also demonstrate that a DNA-only, less than 50-gene next-generation sequencing (sNGS) panel combined with ultra-rapid reflex testing via Idylla Genefusion assay provides a cost-effective testing schema for most lung cancer cases, thereby largely negating the need for upfront *ALK* and *ROS1* fusion testing by FISH. Lastly, we demonstrate the need for additional reflex testing capability on larger DNA and fusion panels for a small subset of lung tumor specimens bearing rare genetic aberrations.

## 2. Materials and Methods

### 2.1. Clinical Specimens

We retrospectively analyzed NGS and *ALK/ROS1* FISH results for all patient samples with primary or metastatic lung adenocarcinoma (*n* = 368) referred to the University of Rochester Medical Center (URMC) molecular diagnostics and cytogenetics laboratories for mutational testing and fusion analysis from July 2021 to June 2022. Clinical data were collected as approved under an Institutional Review Board protocol allowing genomic testing on patients’ tumors.

### 2.2. Sample Collection for Idylla Gene Fusion Assay Validation

Cases of lung adenocarcinoma with known gene rearrangements involving *ALK*, *ROS1*, *RET*, and *MET* exon 14 skipping mutations were identified on the basis of standard-of-care testing using NGS and FISH at the URMC MGP laboratories. Each positive sample served as a negative control for the remaining aberrations, and eight NGS/FISH negative cases were also included. Electronic medical records were reviewed to obtain data regarding tumor characteristics.

### 2.3. Validation of the Idylla Gene Fusion Assay

The Idylla Genefusion Assay (Biocartis, Inc., Mechelen, Belgium) is a cartridge-based, automated multiplex RT-qPCR test designed to detect rearrangements in *ALK*, *ROS1*, *RET* (both for specific translocations and 3′5′ expression imbalance) and *NTRK1/2/3* rearrangements (3′5′ expression imbalance only) and *MET* exon 14 skipping mutations within a span of 3.5 h. Automation is enabled through a cartridge-based system, in which, beginning with the FFPE slide(s), nucleic acid extraction, reverse transcription, amplification, and variant detection occur. Each cartridge contains a single sample and is intended for one-time use. Briefly, following insertion of the scraped FFPE tissue sample into the cartridge, the cartridge is placed into the lysis chamber in the Idylla instrument. Within the lysis chamber, various chemicals and reagents, heat, and high-intensity focused ultrasound enable deparaffinization and cell lysis. Nucleic acids are then purified and concentrated on a solid support, and following elution they are transferred to the PCR chambers for multiplex RT-qPCR using TaqMan PCR and fluorescence-based detection.

Gene fusion detection in the Idylla system is accomplished through detection of fusion-specific amplification and expression imbalances. Proprietary fusion-specific primers amplifying the flanking sequences of the *ALK*, *ROS1*, and *RET* fusion breakpoints are used. *MET* exon 14 skipping variant and wild-type transcripts are identified with dedicated primer and probe sets. To compare the levels of expression, multiple probes are used to compare wild-type expression to the remaining samples. Notably, the assay is only able to detect the gene rearrangements and is unable to identify the specific fusion partner.

Assessment of the limit of detection (LOD) was accomplished using six different cell lines harboring the different mutations. Cell lines were grown and then mixed to reflect the tested ratios (25%, 20%, 10%, and 5%) for *ALK*, *ROS1*, *RET*, and *MET* exon 14 skipping mutations. FFPE cytospin pellets were prepared for testing. See the Supplemental Data for a detailed description of the assay validation.

### 2.4. Performance Comparisons with Reference Methods

NGS and FISH served as reference modalities in all cases. A subset of cases was submitted for testing by a commercial provider (Foundation Medicine, Inc., Cambridge, MA, USA) but the majority of cases were tested using a small, in-house 35-gene NGS (sNGS) assay. Specifically, the URMC solid tumor oncology NGS test uses the DNA-only portion of Oncomine Focus Assay (OFA) (ThermoFisher, Inc., Waltham, MA, USA), which enables single-nucleotide variant (SNV) and small indel detection and interrogates hotspot regions of 35 genes. This panel also possesses an additional, partly overlapping set of 18 genes for copy number variation (CNV) analyses and RNA fusion transcript detection capability for 23 genes. The limit of detection for SNVs and indels is precise and reproducible at 5% allele frequency with 500× coverage. The limit of detection of indels is less than 25 bp. Variants of uncertain origin (germline versus somatic origin) cannot be determined unequivocally based on sequencing tumor samples alone. The data obtained are analyzed with the Ion Reporter^TM^ software for variant classification and the Torrent Suite Analysis plugin for the coverage analysis. The mutation nomenclature is based on a recommendation from the *Catalogue of Somatic Mutations in Cancer* (COSMIC, http://cancer.sanger.ac.uk/cosmic (accessed on 1 July 2023)). Note that only the SNV/small indel detection portion of the OFA assay has been validated for use by our laboratory. The list of genes of the OFA includes: *AKT1* NM_001014431.1, *BRAF* NM_004333.4, *DDR2* NM_006182.2, *ERBB3* NM_001982.3, *FGFR2* NM_000141.4, *GNAQ* NM_002072.4, *IDH2* NM_002168.2, *JAK3* NM_000215.3, *MAP2K1* NM_002755.3., *MTOR* NM_004958.3, *PIK3CA* NM_006218.3, *ROS1* NM_002944.2, *AR* NM_000044.3, *CTNNB1* NM_001904.3, *ERBB2* NM_004448.3, *ESR1* NM_001122740.1, *GNA11* NM_002067.4, *IDH1* NM_005896.2, *JAK2* NM_004972.3, *KRAS* NM_033360.3, *MET* NM_001127500.2, *PDGFRA* NM_006206.5, *RET* NM_020975.4, *ALK* NM_004304.4, *CDK4* NM_000075.3, *EGFR* NM_005228.4, *ERBB4* NM_005235, *FGFR3* NM_000142.4, *HRAS* NM_001130442.1, *JAK1* NM_002227.2, *KIT* NM_000222.2, *MAP2K2* NM_030662.3, *NRAS* NM_002524.4, *RAF1* NM_002880.3, *SMO* NM_005631.4.

FISH was carried out using formalin-fixed, paraffin-embedded tissues with 4 μm sections mounted on positively charged slides. Pretreated slides were incubated in an oven at 60 °C for 1 h, and then processed on a VP2000 (Abbott Molecular, Abbott Park, IL, USA), which includes deparaffinization and protease pretreatment. Slides were hybridized using commercial break-apart probes for *ALK* and *ROS1* (Abbott Molecular, Abbott Park, IL, USA) in IntelliFISH hybridization buffer (Abbott Molecular, Abbott Park, IL, USA) for 2 h at 37 °C after being denatured for 5 min at 73 °C. Slides were washed and counterstained with DAPI (1 µg/mL) (catalog#: 06J49-001, Abbott Molecular, IL, USA). A total of 200 cells was analyzed by two technologists, with each one reading 100 cells. Specimens were considered positive for *ALK* or *ROS1* rearrangement if >15% of the cells demonstrated a positive signal pattern (split ALK or ROS1 signal).

### 2.5. Classification of Mutations

The identified variants were stratified into one of four tiers based on published clinical or laboratory evidence. Tier I includes mutations and fusions that are categorized as variants of strong clinical significance and possess Level A (an FDA-approved therapy and/or included in professional guidelines) or Level B evidence (well-powered studies with expert consensus). Tier II consists of alterations that are classified as variants of potential clinical significance with Level C (FDA-approved therapies for different tumor types or investigational therapies or multiple small published studies with some consensus) or Level D evidence (preclinical trials or a few case reports without consensus). Tier III encompasses mutations that are known as variants of unknown clinical significance, which are not observed at significant allele frequencies in the general population or in specific databases. Finally, Tier IV includes benign or likely benign variants that are observed at significant allele frequencies in the general or specific subpopulation databases.

### 2.6. Statistical Analysis

Data analysis and figures were created using Microsoft Excel, GraphPad 8.3, and CorelDraw 2019 software programs.

## 3. Results

### 3.1. ALK or ROS1 Fusion-Bearing Lung Cancer Cases Are Rare and Do Not Bear Additional SNV, or Small Indel Variants as Determined by the OFA NGS Test

Currently, standard molecular testing of NSCLC at URMC involves FISH testing for *ALK* and *ROS1* fusions, which is performed concomitantly with sNGS for 35 hotspot genes using the ThermoFisher’s Oncomine Focus Assay (OFA) [10]. If these tests do not identify any mutation, recommendations are made for send-out testing for *RET* and other rare fusion transcripts (e.g., for *BRAF* fusions) and for *MET* exon 14 skipping mutation detection at the RNA level.

**Figure 1:**
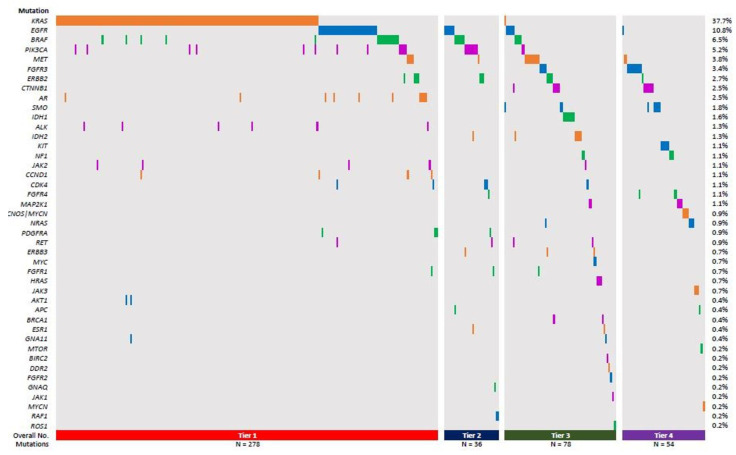
Frequency of different mutations identified by in-house NGS grouped according to Tiers. Total percentage frequencies of the different gene-harboring Tier I-IV variants (depicted by the different colors; Red-Tier 1, Blue-Tier 2, Green–Tier 3, Purple-Tier 4) are listed on the right hand side. The specimens harboring the Tier I–IV variants is depicted by the columns.

To assess the effectiveness of this combination approach, we compiled the results of in-house FISH and sNGS assays for all lung tumors (primary or metastatic) that were submitted for testing during a 1-year time period (July 2021–June 2022), for a total of 368 specimens. We found that only 2.17% (8/368) of lung tumor cases in our cohort harbored an *ALK* fusion, while a mere 0.54% (2/368) possessed a *ROS1* rearrangement caused by FISH, a ratio that is somewhat lower than was reported in a previous study [11]. In contrast, in the *ALK* and *ROS1* fusion-negative specimens, the OFA sNGS panel identified NSCLC-relevant SNVs and indels in nearly 80% (293/368) of cases, indicating that at least one oncogenic or likely oncogenic variant or a variant of uncertain significance (VUS) was identified. One specimen was positive for both a *ROS1* fusion and a pathogenic *KRAS* mutation. In our cohort, lung tumors bearing oncogenic Tier I *KRAS* (~40%) mutations were found most commonly, followed by Tier I–III *EGFR*, *BRAF*, and *PIK3CA* variants and then a number of less common Tier I–IV variants (Figure 1). Furthermore, about 278 (76%) cases in our cohort harbored at least one Tier 1 mutation or more, 36 cases (9.8%) had at least Tier II mutations, 78 (21.2%) had Tier III, while 54 (14.7%) had Tier IV mutations (Figure 1). These data demonstrate that sNGS is a high-yield frontline testing modality, while FISH for *ALK* and *ROS1* fusion should be reserved for DNA-variant negative cases in the workup of NSCLCs.

### 3.2. Validating the Idylla Gene Fusion Assay

Recently, the BioCartis Idylla Genefusion assay, a rapid testing modality with a turn-around time (TAT) of less than 4 h and technologist hands-on time < 5 min has been introduced to identify targetable, NSCLC-related fusion transcripts (*ALK*, *ROS1*, *RET*, *NTRK1-3*) and *MET* exon 14 splice mutations [12,13,14]. Therefore, to potentially complement sNGS, we validated the Idylla Genefusion assay for *ALK*, *ROS1*, and *RET* rearrangements and *MET* exon 14 skipping variant detection using 34 tumor samples that were previously orthogonally tested via FISH and NGS. We assessed 24 surgical resections/biopsies and 10 cytological blocks paraffin-embedded tumor samples with tumor cellularity between 10 and 90% (Table 1 and Supplemental Table S1).

Of the tumor samples, 13 harbored *ALK* fusion mutations, 5 harbored *ROS1* fusion mutations, 2 harbored *RET* fusion mutations, 6 contained *MET* exon14 skipping mutations, and 8 were negative controls (Table 2). Overall, concordance was highest when detecting *RET* fusions (100%, *n* = 2) and *MET* exon 14 skipping mutations (100%, *n* = 6), somewhat lower for *ALK* fusions (92%, *n* = 13), and lowest for *ROS1* fusions (75%, *n* = 5). Detection of fusions via fusion-specific (FS) primers and expression imbalance (EI) by the Idylla Genefusion cartridge demonstrated high-level sensitivity (92.3%) and specificity (100%) (Table 2).

These samples were also tested on different instruments by different technologists on different days to demonstrate inter-assay reproducibility (Supplementary Figure S1). Both FS- and EI-based detection demonstrated high reproducibility with a range of specific fusion cycles among triplicate runs of <1.5 and a range of ΔCQ values among triplicate runs of <1 cycle. Using cell lines harboring various targetable kinase gene fusions, the limit of detection was determined to be 10% tumor cell content for ALK, ROS1, and RET rearrangements (Supplementary Figure S1).

### 3.3. Testing the Diagnostic Utility of Idylla Genefusion Assay

**Figure 2:**
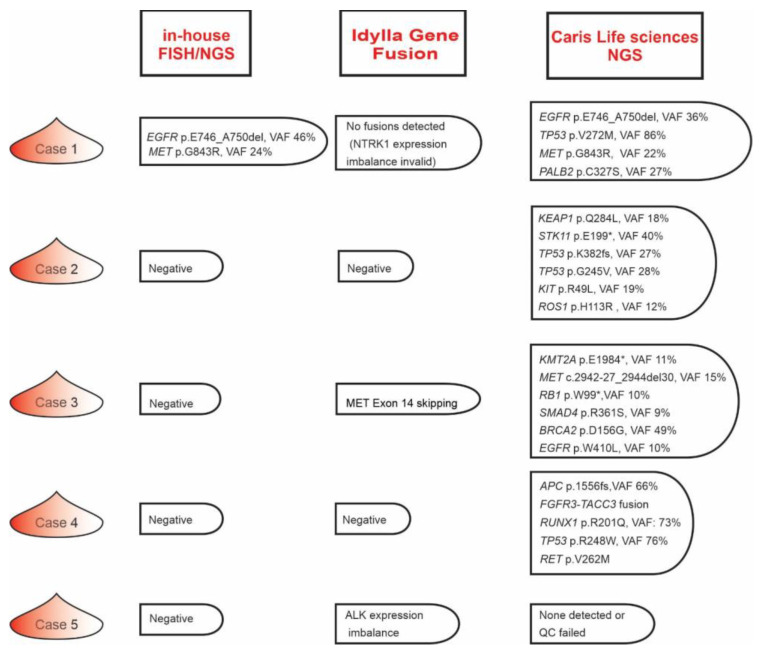
Results of in-house molecular tests compared to a commercial test result. The results of molecular tests involving in-house FISH, sNGS and Idylla gene fusion assays were compared to Caris, Inc.’s MI Tumor Seek Hybrid^TM^ test, which provides combined NGS-based whole exome (WES) and transcriptome sequencing (WTS) profiling on FFPE samples, on five selected specimens.

While NGS-based detection of genomic mutations on the OFA sNGS panel identified Tier I-III variants in a high percentage of cases, more than 20% of specimens lacked genetic variants on the OFA panel and were also *ALK/ROS1* rearrangement negative according to FISH. Therefore, we wished to assess the Idylla Genefusion assay’s utility in these cases. To this end, we selected four in-house NGS/FISH negative cases (Figure 2, cases 2–5) together with one case (Figure 2, case 1) which harbored an sNGS-detected oncogenic *EGFR* p.E746_A750del (Tier I) and *MET* p.G843R (Tier III) VUS with variant allele frequencies (VAF) of 46% and 24%, respectively.

The Idylla Genefusion assay yielded negative results for three of these cases (cases 1, 2, and 4) but identified a MET exon 14 skipping mutation in case 3 (Figure 2). Another case (case 5) showed *ALK* expression imbalance, potentially uncovering a rare *ALK* fusion that was not represented on the specific fusion partner portion of the Idylla Genefusion assay and which was also negative according to FISH.

### 3.4. Fine Tuning the Lung Cancer Molecular Testing Blueprint

While the Idylla Genefusion assay efficiently complemented our in-house OFA sNGS panel, in a small subset of NSCLC cases, we failed to identify any genetic aberrations using the three in-house testing modalities (FISH, sNGS, Idylla Genefusion assays). Thus, to further clarify the mutational profile of these five cases, we subsequently sent out 10–16 FFPE slides for testing by Caris, Inc.’s MI Tumor Seek Hybrid^TM^ test, which provides combined NGS-based whole-exome (WES) and transcriptome sequencing (WTS) profiling on FFPE samples. Except in case 5, in which the Caris test failed at the WES level, a number of genetic variants were uncovered in the three cases in which the OFA sNGS test identified no variants (Figure 2, cases 2, 3, 4). In case 1, the MI Tumor Seek Hybrid^TM^ test confirmed the OFA sNGS detected *EGFR* p.E746_A750del and *MET* p.G843R variants and identified two additional VUSs. Notably, all sNGS negative cases harbored mutant gene variants with prognostic significance at the WES level but only one specimen (case 4) harbored a potentially actionable *FGFR3::TACC3* fusion that defines an unique molecular subtype of NSCLC and that may be amenable to targeted therapy [15,16]. The TAT for the MI Tumor Seek Hybrid^TM^ test for the five cases varied from 10 to 21 days and required 10–16 FFPE slides. Thus, for OFA sNGS plus Idylla Genefusion negative NSCLC cases, prompt send-out testing for combined WES/WTS testing is warranted if a sufficient amount of material remains available.

## 4. Discussion

Molecular testing for lung cancer is well established, but the guidelines of how to provide optimal patient care while also providing rapid, cost-effective, insurance-reimbursed testing modalities are yet to be established. In this study, we demonstrate that a small (<50 gene) DNA-only NGS (sNGS) panel combined with ultra-rapid reflex testing for relatively common fusion transcripts using the Idylla Genefusion assay provides one such approach for efficient tumor profiling in most cases of advanced NSCLC.

We found that after obtaining negative results on our in-house sNGS panel, subsequent testing for NSCLC relevant fusion transcripts using the Idylla Genefusion assay was rapid and easy to perform with a TAT of <4 h. Following validation of the assay using cases of lung adenocarcinoma with known gene rearrangements involving *ALK*, *ROS1*, *RET*, and *MET* exon 14 skipping mutations, we identified a *MET* exon 14 skipping mutation in one NGS-negative case and *ALK* expression imbalance in another, of which the *MET* exon 14 skipping mutation was subsequently confirmed by WES/WTS NGS via a commercial assay. In agreement with this, several studies performed recently have shown that the Idylla Genefusion testing has high sensitivity, specificity, and overall high concordance with the reference methods [13,14,17,18]. This *MET* exon 14 skipping mutation could not be detected by our in-house DNA-based NGS panel due to the absence of this region on *MET* amplicons present in the OFA sNGS panel.

We also found that of 368 samples tested for *ALK* and *ROS1* rearrangements via FISH, a mere 2.8% (6/368) were *ALK*-fusion positive and only 0.5% (2/368) were *ROS1*-fusion positive. In contrast, we detected Tier I-III mutations by OFA NGS in nearly 80% (294/368) of samples. These data imply that *ALK* and *ROS1* rearrangement detection by FISH should not be offered as frontline testing, and that in lung tumor specimens (lung primary and metastases) sNGS-detectable SNV and indel variants are common, but only rarely co-occur with *ALK*, *ROS1*, and *RET* fusion variants or *MET* exon 14 skipping mutations.

Our results are comparable to those previously reported by Jordan et al. using MSK-IMPACT. Consistent with the MSK-IMPACT study, mutant *EGFR* and *KRAS* were the most commonly identified oncogenic drivers in our cohort and were found to be mutually exclusive. In contrast, *ALK*, *ROS1*, *RET*, and *MET* exon 14 skipping alterations were infrequent in our cohort, comparable to the 1.7–3.8% frequency reported by Jordan et al. [11]. Taken together, this suggests that fusion transcripts in DNA variant-bearing lung cancers are rare. Therefore, we propose that fusion transcript testing should be reserved for sNGS panel variant-negative cases (Figure 2).

Moreover, reflex Idylla Genefusion assay testing is rapid and feasible and provides an ideal complement for sNGS-negative samples, allowing for direct incorporation of the results into sNGS reports prior to case sign-out. Rarely, FISH-detected *ALK* or *ROS1* fusions were not detected by the Idylla Genefusion assay, perhaps due to an unusual translocation pattern. In such instances and in cases in which the tumor cellularity is below 10%, reflexing OFA sNGS and Idylla Genefusion-negative cases for *ALK* and *ROS1* fusion detection by FISH and/or IHC may be necessary. Finally, the rare sNGS panel plus Idylla Genefusion negative NSCLC cases should be considered for rapid send-out WES/WTS profiling to a reputable commercial vendor with a reasonable TAT.

This study and the diagnostic workflow proposed have some limitations. Detection of expression imbalance only, as was seen in case 5, or no identification of mutation by NGS or Idylla genefusion testing warrants additional testing using an orthogonal method. Though the Caris MI Tumor Seek Hybrid^TM^ test was able to identify the genetic alterations in almost all the samples, the average turnaround time for the results was about 14 days. Furthermore, as previously demonstrated, the larger NGS panels identify more variants; however, most of them do not have an impact on patient management [19]. Comparative analysis between the smaller NGS panels and larger panels show that the smaller panels detect at least 90% of the mutations and are nearly as informative as the larger panels when identifying actionable genetic alterations [19].

Another important issue to consider is the cost of these different diagnostic tests. Currently, for URMC, the reagent cost per sample for the OFA sNGS assay is approximately USD 380, while that of the Idylla Genefusion assay is approximately USD 640 per cartridge. The send-out testing cost of the Caris MI Tumor Seek Hybrid^TM^ test (per report) is currently approximately USD 3500. Thus, the suggested testing modality scheme of sNGS > Genefusion > sendout is also price competitive, while avoiding significant upfront expense for large-capacity NGS instruments (and their yearly maintenance fee), which represents a significant issue for smaller and midsize academic molecular laboratories.

Finally, the TAT of the OFA sNGS panel is 10–14 days, making it suboptimal for NSCLC molecular diagnostics. Therefore, we plan to replace our current OFA sNGS assay with the Oncomine Precision Assay (OPA) on the Genexus sNGS platform, which would only require 30–60 min of hands-on technologist time and a much faster TAT of 3–5 days. The reagent cost per sample for this assay would be approximately USD 420. Additionally, the OPA contains both DNA and RNA fusion sequencing portions and allows simultaneous extraction of both DNA and RNA from the submitted specimens. Future studies will evaluate whether the utilization of the OPA in its full DNA/RNA sequencing mode is faster and/or more cost effective for NSCLC molecular diagnostics compared to the current proposed modality of coupled DNA-only sNGS and reflex Idylla Genefusion testing.

## Figures and Tables

**Table 1:** Clinicopathologic characteristics of samples evaluated using the Idylla testing.

Sample Type	
Surgical resection/biopsy	24
Cytology cell blocks	10
**Primary Site**	
Lung	15
Lymph node	7
Thyroid	6
Bone	1
Ovary	1
Brain	1
Pleural fluid	1
Diaphragmatic implant	2
**Tumor Cellularity**	
>70%	16
>50–70	8
>40–50	3
>30–40	1
>20–30	1
>10–20	4
0–10	1

**Table 2:** Idylla Genefusion performance. FS—fusion specific; EI—expression imbalance.

Kinase Gene	FISH/NGS Confirmed		Idylla				Overall Concordance
	Positive	Negative	Positive	Negative	FS	EI	
*ALK* fusions	13	8	12	9	12	9	92% (12/13)
*RET* fusions	2	8	2	8	2	2	100% (2/2)
*ROS1* fusions	5	8	4	9	3	2	80% (4/5)
*MET* exon 14 skipping	6	8	6	8	6	N/A	100% (6/6)

## Data Availability

The consent documentation signed by the patients do not expressly allow submission of full sequencing data (FASTQ, BAM/BAI, VCF) to external data repositories.

## Supplementary Materials

The following supporting information can be downloaded at: https://www.mdpi.com/article/10.3390/genes14081551/s1, Figure S1: Reproducibility and limit of detection for the Idylla GeneFusion assay; Table S1: Detailed data on Idylla Genefusion assay validation.
