# Supplementary material for: Efficient Lung Cancer Molecular Diagnostics by Combining Next Generation Sequencing with Reflex Idylla Genefusion Assay Testing
Source: Genes (Basel). 2023 Jul 28;14(8):1551. doi: 10.3390/genes14081551 (PMC10454377; doi:10.3390/genes14081551)
Supplement: Supplementary file 1 [file genes-14-01551-s001.zip › genes-2519993-supplementary-Figure S1.pdf]

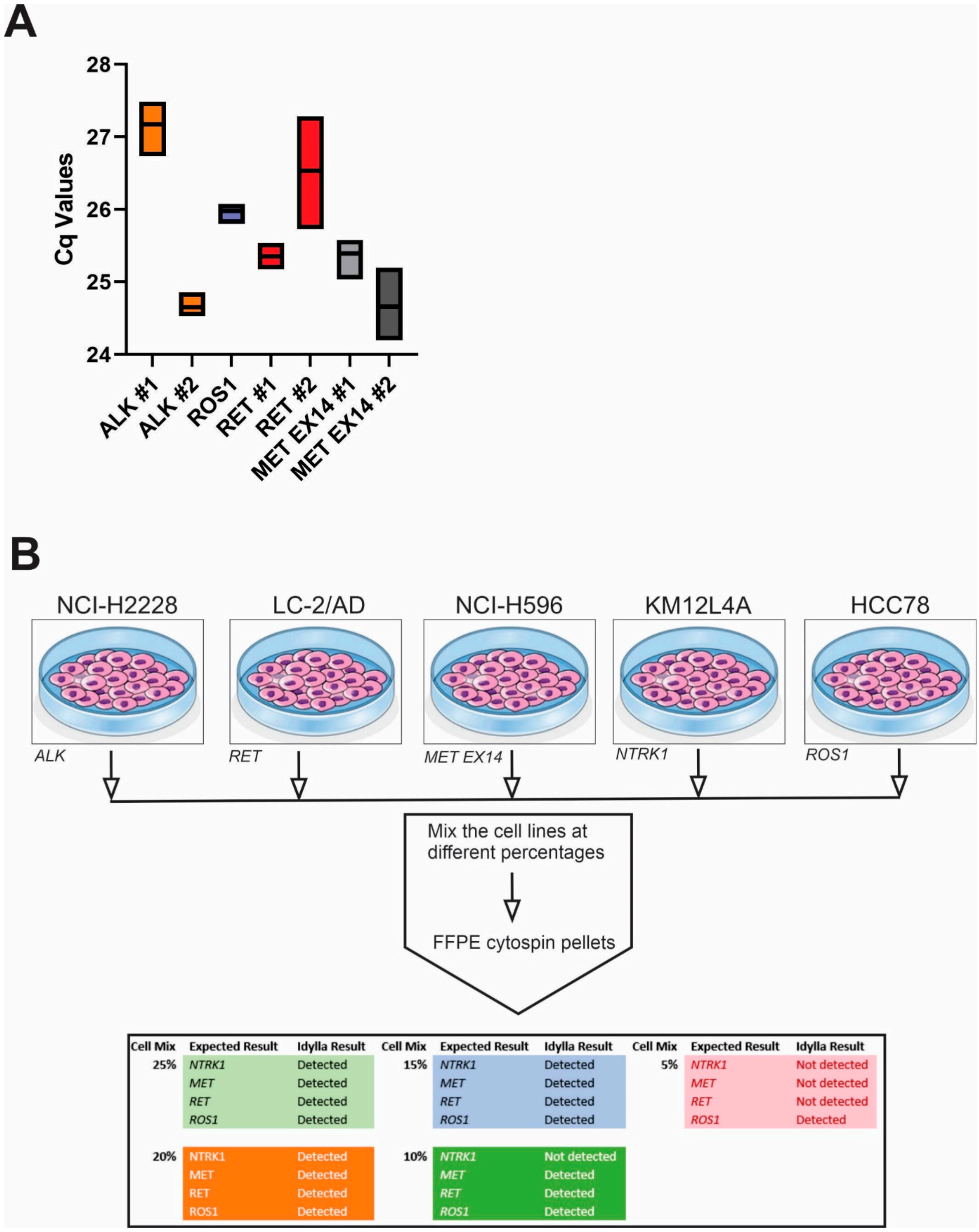

Supplementary Figure S1: Reproducibility and Limit of detection for the Idylla GeneFusion assay. (A) Precision & Reproducibility of results on the Idylla GeneFusion assay. Different clinical samples were run by different technologist on different instruments and different days. Cq values of two samples obtained by the different technologist are shown in the graph; (B) Limit of detection of the Idylla GeneFusion assay. The study was performed using six different cell lines harboring different gene fusions. Cell lines were grown and expanded and then mixed to reflect the tested ratios (25%, 20%, 10% and 5%) for *ALK, ROS1, RET* and *MET* exon 14 skipping mutations. FFPE cytospin pellets were prepared for testing and run on the Idyalla.
